# Optimization of environmental DNA extraction and amplification methods for metabarcoding of deep-sea fish

**DOI:** 10.1016/j.mex.2021.101238

**Published:** 2021-01-23

**Authors:** Masaru Kawato, Takao Yoshida, Masaki Miya, Shinji Tsuchida, Yuriko Nagano, Michiyasu Nomura, Akinori Yabuki, Yoshihiro Fujiwara, Katsunori Fujikura

**Affiliations:** a Marine Biodiversity and Environmental Assessment Research Center (BioEnv), Research Institute for Global Change (RIGC), Japan Agency for Marine-Earth Science and Technology (JAMSTEC), Yokosuka, Kanagawa, Japan; b Natural History Museum and Institute, Chiba, Chuo-ku, Chiba, Japan; c DHC Corporation, Japan

**Keywords:** Pumped deep-sea water, eDNA yield, MiFish, PCR efficiency

## Abstract

Analyses of environmental DNA (eDNA) from macroorganisms in aquatic environments have greatly advanced in recent years. In particular, eDNA metabarcoding of fish using universal PCR primers has been reported in various waters. Although pumped deep-sea water was used for eDNA metabarcoding of deep-sea fish, conventional methods only resulted in small amounts of extracted eDNA and subsequent few or no PCR amplicons. To optimize eDNA metabarcoding of deep-sea fish from pumped deep-sea water, we modified conventional procedures of eDNA extraction and PCR amplification. Here, we propose a modified eDNA extraction method, in which a filter used for eDNA sampling was shredded and incubated in microtubes for efficient lysis of eDNA sources. Total eDNA yield extracted using the modified protocol was approximately six-fold higher than that extracted by the conventional protocol. The PCR enzyme Platinum SuperFi II DNA Polymerase successfully amplified a target region of fish universal primers (MiFish) from trace amounts of eDNA extracted from pumped deep-sea water and suppressed nonspecific amplifications more effectively than the enzyme used in conventional methods. Approximately 93% of the sequence reads acquired by next generation sequencing of these amplicons were derived from fish. The improved procedure presented here provided effective eDNA metabarcoding of deep-sea fish.

• A modified eDNA extraction protocol, in which a filter was shredded and incubated in microtubes, increased eDNA yields extracted from pumped deep-sea water over the conventional method.
• The PCR enzyme Platinum SuperFi II DNA polymerase improved the amplification efficiency of trace amounts of MiFish objectives in eDNA extracted from pumped deep-sea water with suppressing nonspecific amplifications.
• The use of Platinum SuperFi II DNA polymerase for eDNA metabarcoding using MiFish primers resulted in the acquisition of abundant sequence reads of deep-sea fish through next generation sequencing.

## Graphical abstract

**Figure:**
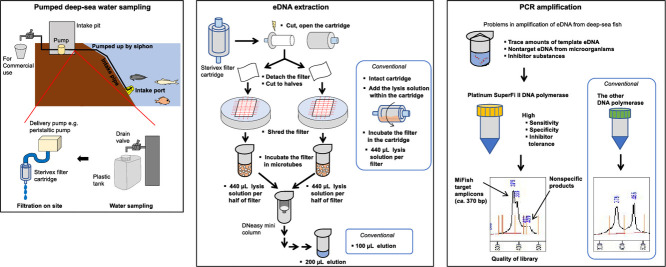

Specifications table

**Table:** 

Subject Area	Agricultural and Biological Sciences
More specific subject area	Deep-sea fish biodiversity research using eDNA metabarcoding
Method name	Methods of environmental DNA extraction and PCR amplification for deep-sea fish
Name and reference of original method	• M. Miya, T. Minamoto, H. Yamanaka, S.I. Oka, K. Sato, S. Yamamoto, T. Sado, H. Doi, Use of a Filter Cartridge for Filtration of Water Samples and Extraction of Environmental DNA, J. Vis. Exp. (117) (2016). • M. Miya, Y. Sato, T. Fukunaga, T. Sado, J.Y. Poulsen, K. Sato, T. Minamoto, S. Yamamoto, H. Yamanaka, H. Araki, M. Kondoh, W. Iwasaki, MiFish, a set of universal PCR primers for metabarcoding environmental DNA from fishes: detection of more than 230 subtropical marine species, R. Soc. Open. Sci. 2(7) (2015). • M. Miya, T. Sado, DNA extraction from a filter cartridge. Pages 31–42 in Environmental DNA sampling and experimental manual version 2.1. Ed. by eDNA Methods Standardization Committee, The eDNA Society, Otsu, Japan. (2019). • M. Miya, T. Sado, Multiple species detection using MiFish primers. Pages 55–92 in Environmental DNA sampling and experimental manual version 2.1. Ed. by eDNA Methods Standardization Committee, The eDNA Society, Otsu, Japan. (2019).
Resource availability	Information on all resources needed to reproduce this procedure is included in the present article.

## Method details

### Background

Since the analysis of environmental DNA (eDNA) of vertebrate in an aquatic environment was reported in 2008 [1], eDNA analyses aimed at evaluating fish ecology, such as species presence/absence, diversity inventory, biomass estimate, and detection of introduced species, have been applied to various aquatic environments, including ponds/lakes [2], [3], [4], [5]–6], rivers [7], [8], [9], [10]–11], and oceans [12], [13], [14], [15], [16], [17], [18]. A strong driving force behind the advances in eDNA analyses of fish was the development of the "MiFish" universal PCR primers for eDNA metabarcoding of fish [19], as described in the review by Miya et al. (2020). A detailed protocol for eDNA analyses of fish, including filtration of water samples and extraction of eDNA from filters, has been recently developed [20]. The "Environmental DNA Sampling and Experiment Manual" (hereinafter referred to as "eDNA manual") for standardizing eDNA research is open to the public from The eDNA Society and has contributed to eDNA analyses in fish studies worldwide [21]. Nevertheless, eDNA analyses aimed at deep-sea fish communities have been limited [22]. There have been several difficulties in conducting eDNA analyses using deep-sea water, such as limited sampling opportunities, limited water volumes from sampling devices, limited eDNA amounts from deep-sea fish due to the low biomass, and accumulation of PCR inhibitors and nontarget eDNA due to the filtration of large volumes of water. It is thus necessary to overcome these difficulties for better understanding of the deep-sea fish communities using eDNA information.

There are more than 10 facilities for pumping up deep-sea water for commercial and research purposes in Japan. The pumped deep-sea water is more easily accessible to researchers than the deep-sea water collected using water sampling devices operated from vessels. Therefore, we used it as a source of deep-sea fish eDNA in this study. Although there have been several reports and reviews concerning the procedures of eDNA extraction and amplification for metabarcoding of fish from various waters [23], [24], [25], [26], [27]–28], no optimized method for eDNA metabarcoding of deep-sea fish using pumped deep-sea water has been reported yet. In our preliminary eDNA metabarcoding experiments using pumped deep-sea water as an eDNA source, we could not successfully extract eDNA, nor amplify the target region of MiFish PCR primers using previously published methods [19,20]. This was probably due to trace amounts of eDNA sources in water samples and the low concentration of fish eDNA available as template for PCR amplifications. Thus, to enable eDNA metabarcoding of deep-sea fish from pumped deep-sea water, we optimized eDNA extraction and PCR amplification of the 12S rRNA gene targeted by the MiFish primer set.

### Water sampling and filtration

Pumped deep-sea water was collected at the DHC deep-sea water pumping facility (Akazawa, Shizuoka, Japan) and Shizuoka prefectural deep-sea water pumping facility (Yaizu, Shizuoka, Japan), both of which are located at the Pacific coast of central Japan facing Sagami Bay and Suruga Bay, respectively. The deep-sea water was pumped up off Akazawa at a depth of 800 m, and off Yaizu at a depth of 397 m. The collected water was immediately filtered on-site. We used an enclosed type filter, Sterivex-HV Pressure Filter Unit (0.45 µm pore size, PVDF membrane, gamma irradiated, sterile) (Merck KGaA, Darmstadt, Germany), which was validated to be used for the filtration of a large amount of water in the previous study [20]. The volume of each filtrated pumped deep-sea water sample was determined according to the clogging condition of each Sterivex filter (*ca*. 20 to 30 L at Akazawa, *ca*. 10 L at Yaizu). The number of filtered samples used for validation experiments in this study is described in the "Method validation" section. After filtration, each Sterivex filter cartridge was filled with 2.0 mL of RNAlater (Thermo Fisher Scientific, Waltham, MA, USA) to prevent digestion of the collected eDNA, as previously described [29]. Filter cartridges were kept in freezers at -30 °C until eDNA extraction.

### eDNA extraction

To improve eDNA yield from each filter cartridge, we designed a modified eDNA extraction protocol using a commercial DNeasy Blood and Tissue Kit (QIAGEN, Valencia, CA, USA), which is based on the conventional protocol described by Miya et al. (2016), as described below (also see the graphical abstract). Any precautions against contamination during eDNA extraction were conducted according to the eDNA manual [30]. Differences among protocols are highlighted in Table 1.

**Table 1:** Differences in procedures of eDNA extraction between modified and conventional protocols.

Step / Treatment	modified protocol	conventional protocol
*Before lysis*		
Filter cartridge treatment	• Cut the outlet connection of the cartridge and divide into the filter unit and the housing	• None
Filter treatment	• Detach the filter from the filter unit • Shred the filter to small fragments	• None
*Lysis*		
Solution	• 440 µL PBS, 400 µL Buffer AL, 40 µL proteinase K, per filter	• 220 µL PBS, 200 µL Buffer AL, 20 µL proteinase K, per filter
Lysis manner	• Add the lysis solution into two microtubes • Incubate all filter fragments in two microtubes with occasional shaking	• Add the lysis solution into the intact filter cartridge • Incubate the filter in the filter cartridge with rotary shaking
Incubation	• 56 °C for 2 h	• 56 °C for 20 min
*Elution from DNeasy column*		
Elution buffer volume	• 200 µL per filter	• 100 µL per filter

### Newly required tools in this modified eDNA extraction protocol

• Tube cutter (TRUSCO tube cutter; TRUSCO Nakayama, Tokyo, Japan)
• Sterile scalpel (KAI scalpel; Kai Corp., Tokyo, Japan)
• Sterile petri dish
• Sterile sharp tweezer

### Protocol

1. Transfer a Sterivex filter cartridge from a freezer to the lab bench and thaw RNAlater in the cartridge at room temperature (15–25 °C).
2. Aspirate RNAlater from the outlet to the extent possible, using a suitable vacuum pump with luer fitting.
3. Transfer the Sterivex filter cartridge to a sterile petri dish.
4. Cut the outlet connection of the cartridge using a tube cutter, divide the cartridge into the filter unit and the housing, and place the filter unit on the other petri dish.
5. Using a sterile scalpel, cut the filter along the edges and vertical lines, and divide the filter in half. Detach and transfer a half of the filter on the other petri dish, and store the other half on the filter unit. Be careful not to dry it out.
6. Cut the transferred filter quickly and carefully into 32 equal-sized fragments on the petri dish using a scalpel and tweezer.
7. Using the tweezer, retrieve all filter fragments into a 2.0 mL microtube filled with a lysis solution containing 220 µL PBS (-), 200 µL Buffer AL*, and 20 µL proteinase K*.
8. Close the lid of the microtube tightly and mix thoroughly by shaking the tube by hand.
9. Transfer the remaining filter from the filter unit to a petri dish and cut into 32 fragments as described in Step 6.
10. Repeat Steps 7 and 8. Two 2.0 mL microtubes with filter fragments are ready for further operations.
11. Place the microtubes in an incubator and incubate at 56 °C for 2 h with occasional shaking.
12. After incubation, centrifuge the microtubes at 15,000 × *g* for 1 min.
13. Transfer the supernatant carefully from each microtube into a new single 2.0 mL microtube. Thus, it includes approximately 880 µL solution.
14. Then, add 400 µL ethanol (96–100%) to the 2.0 mL microtube and mix thoroughly by pipetting.
15. Transfer up to 700 µL of the solution into a DNeasy mini spin column* placed in a collection tube*.
16. Centrifuge the column at 6000 × *g* for 1 min.
17. Discard the flow-through, and repeat Steps 15 and 16, until all the DNA solution passes through the column.
18. Discard the collection tube and place the column in a new collection tube*.
19. Subsequently, add 500 µL Buffer AW1* to the column and centrifuge the column at 6000 × *g* for 1 min.
20. Discard the collection tube and place the column in a new collection tube*.
21. Add 500 µL Buffer AW2* to the column and centrifuge the column at 20,000 × *g* for 3 min.
22. Discard the flow-through, place the column back in the empty collection tube, and centrifuge the column again at 20,000 × *g* for 1 min.
23. Discard the collection tube and place the column in a new 1.5 mL or 2.0 mL microtube.
24. Add 200 µL Buffer AE* directly onto the DNeasy membrane. Incubate at room temperature (15–25 °C) for 1 min and then centrifuge at 6000 × *g* for 1 min.
25. Optionally, place the column in a new 1.5 mL or 2.0 mL microtube and repeat the elution Step 24.
26. Discard the column. Cap the tube, confirm the label, and store the eluted eDNA solution in a freezer until use.

* Reagents and expendables included in the DNeasy Blood and Tissue Kit.

### First PCR amplification for eDNA metabarcoding

We used a newly released PCR enzyme, Platinum SuperFi II DNA polymerase (Thermo Fisher Scientific, Waltham, MA, USA) (hereinafter referred to as "SuperFi II") in the first PCR for eDNA metabarcoding of deep-sea fish from pumped deep-sea water. The decision to use SuperFi II instead of KAPA HiFi HotStart DNA polymerase (Kapa Biosystems, Wilmington, MA, USA) (hereinafter referred to as "KAPA"), which was used in previous eDNA studies [11,^16^,^18^,^19^,^31^,32] and introduced in the eDNA manual [33], was based on its high sensitivity, inhibitor tolerance, and specificity in the amplification reaction, as described in the manufacturer's instruction.

### A newly required tool in this modified PCR

• Platinum SuperFi II DNA polymerase (Thermo Fisher Scientific, Waltham, MA, USA)

### Primers for first PCR

We used the fish universal PCR primers "MiFish", which were developed by Miya et al. (2015) for eDNA metabarcoding of fish. These primers were designed for amplification of a hypervariable region (*ca*. 170 bp) of the 12S rRNA gene, and were confirmed to be versatile across a diverse range of fish, including deep-sea species [19]. The first PCR we performed was a multiplex PCR using two primer pairs mixed in equimolar amounts, that is, MiFish-MIX-F including MiFish-U-F and MiFish-E-F and MiFish-MIX-R including MiFish-U-R and MiFish-E-R. In particular, the MiFish-U pairs are known to be universal primers for ray-finned fish, whereas the MiFish-E pairs are primers optimized for elasmobranches [19]. Primer sequences are provided below: Nearly half of the nucleotides are adapters for the second PCR, with the gene-specific sequence of MiFish being followed by six random hexamers (N).

MiFish-MIX-F (forward)

MiFish-U-F: 5′-ACACTCTTTCCCTACACGACGCTCTTCCGATCT-NNNNNN-GTCGGTAAAACTCGTGCCAGC-3′

MiFish-E-F: 5′-ACACTCTTTCCCTACACGACGCTCTTCCGATCT-NNNNNN-GTTGGTAAATCTCGTGCCAGC-3′

MiFish-MIX-R (reverse)

MiFish-U-R: 5′-GTGACTGGAGTTCAGACGTGTGCTCTTCCGATCT-NNNNNN-CATAGTGGGGTATCTAATCCCAGTTTG-3′

MiFish-E-R: 5′-GTGACTGGAGTTCAGACGTGTGCTCTTCCGATCT-NNNNNN-CATAGTGGGGTATCTAATCCTAGTTTG-3′

### PCR conditions

All precautions taken against contamination were basically according to the instructions of both Miya et al. (2015) and the eDNA manual [33]. The first PCR was conducted using eight technical replicates per eDNA template, as described in previous studies [5,28] and the eDNA manual [33]. The composition of reagents was almost identical to the SuperFi II user guide provided by the manufacturer, with minor modifications, as shown in Table 2. Thermal cycling was performed in accordance with the manufacturer's recommendation, and was as follows: 98 °C for 30 s for initial denaturation, followed by 38 cycles of denaturation at 98 °C for 10 s, annealing at 60 °C for 10 s, and extension at 72 °C for 30 s; with a final extension at 72 °C for 5 min. The annealing temperature was set at 60 °C, because SuperFi II provides universal annealing temperature regardless of the primer sequences (see the SuperFi II user guide). The number of cycles was determined to be 38 based on a preliminary experiment (Supplementary Fig. 1).

**Table 2:** Composition of PCR reagents in Platinum SuperFi II DNA Polymerase reaction for amplification of MiFish objectives using eDNA extracted from pumped deep-sea water. ^*1^: This reaction includes 0.3 µM of MiFish-U and MiFish-E. ^⁎2^: Volumes vary depending on concentrations used.

Reagent components	Final concentration	Volume in 12 µL reaction
5 × SuperFi II Buffer	1 ×	2.4 µL
dNTPs mixture	200 µM each	—*^2^
MiFish-Mix-F (Forward primer)	0.6 µM*^1^	—*^2^
MiFish-Mix-R (Reverse primer)	0.6 µM*^1^	—*^2^
Extracted eDNA	< 10 ng/µL	4 µL
Platinum SuperFi II DNA Polymerase	1 ×	0.24 µL
Nuclease-free sterile water	—	to 12 µL

### Library preparation, next generation sequencing (NGS), and data processing

After the first PCR, subsequent procedures of paired-end library construction and NGS were performed by Bioengineering Lab. Co., Ltd (Sagamihara, Japan), as follows: After purification of the first PCR products using AMPure XP (Beckman Courter, Brea, CA, USA), the second tailed PCR was conducted using primers with appropriate unique index sequences. The reaction was performed in a 10 µL mixture containing 1× Ex Taq buffer (Takara Bio, Shiga, Japan), 0.2 mM of each dNTP, 0.5 µM forward primer, 0.5 µM reverse primer, 0.5 U Ex Taq (Takara Bio, Shiga, Japan), and 2.0 µL of the first PCR product. Thermal cycling was performed as follows: 94 °C for 2 min for initial denaturation, followed by 12 cycles of denaturation at 94 °C for 30 s, annealing at 60 °C for 30 s, and extension at 72 °C for 30 s; with a final extension at 72 °C for 5 min. PCR products were purified using AMPure XP, and then the paired-end sequence libraries were completed. Libraries were sequenced using Illumina Miseq (Illumina, San Diego, CA, USA) under 2 × 300 bp conditions. Raw paired-end sequence reads were processed and analyzed in MitoFish (http://mitofish.aori.u-tokyo.ac.jp/), which is a freely available database of fish mitochondrial genomes with an analysis pipeline for metabarcoding of fish, named MiFish Pipeline [34,35]. Using the MiFish Pipeline, quality check of sequences, tail trimming, paired-end read assembly, removal of unreliable sequences and primer sequences, read clustering, and BLASTN searches were performed according to default settings.

## Method validation

### Comparison of eDNA yields between extraction protocols

We compared the total eDNA yields between extraction protocols, that is, the conventional protocol [20] and the modified protocol presented in this study. We used Akazawa samples for this comparison because total eDNA yields from these samples have been quite low, requiring improved strategies to increase eDNA yields. Filtration volume in each sample was 20 or 30 L (Table 3). Three filters were used for the conventional extraction protocol, and four filters were used for the modified extraction protocol (Table 3). Concentrations of extracted eDNA were measured using a Qubit dsDNA HS assay Kit with a Qubit fluorometer (Life Technologies, Carlsbad, CA, USA). DNA concentrations of each eDNA extracted from a filter were converted to eDNA yields in 1 L of filtered water as follows: (measured DNA concentrations (ng/µL) × volume of eDNA solutions (µL))/filtration volume (L). Converted values were used for comparison (Table 3). The eDNA yields extracted using our new protocol were approximately six-fold greater than those extracted using the conventional protocol (Table 3). This result clearly showed that the present extraction protocol was much more successful in producing a total eDNA yield from pumped deep-sea water samples compared with the conventional method. This was probably due to the efficient lysis and protein digestion of filtered eDNA sources following the immersion of filter fragments in a sufficient volume of lysis buffer within the microtubes, and the occasional shaking.

**Table 3:** Extracted eDNA yields from pumped deep-sea water collected at Akazawa using two different protocols. AK2-1, AK2-7, AK2-8, AK2-10, AK2-11, AK1-16, and AK1-17 represent sample numbers.

Filter No.	Filtration volume (L)	Protocol	eDNA elution volume (µL)	Extracted eDNA concentration (ng/µL)	Total eDNA yield (ng)	eDNA yield per 1 L of filtered water (ng)
AK2-7	20	modified	200	13.0	2600	130.0
AK2-8	20	modified	200	13.4	2680	134.0
AK2-10	20	modified	200	14.4	2880	144.0
AK2-11	20	modified	200	15.5	3100	155.0
AK2-1	20	conventional	100	3.8	380	19.0
AK1-16	30	conventional	100	6.8	680	22.7
AK1-17	30	conventional	100	8.1	810	27.0

### PCR efficiency for metabarcoding of fish

**Fig. 1:**
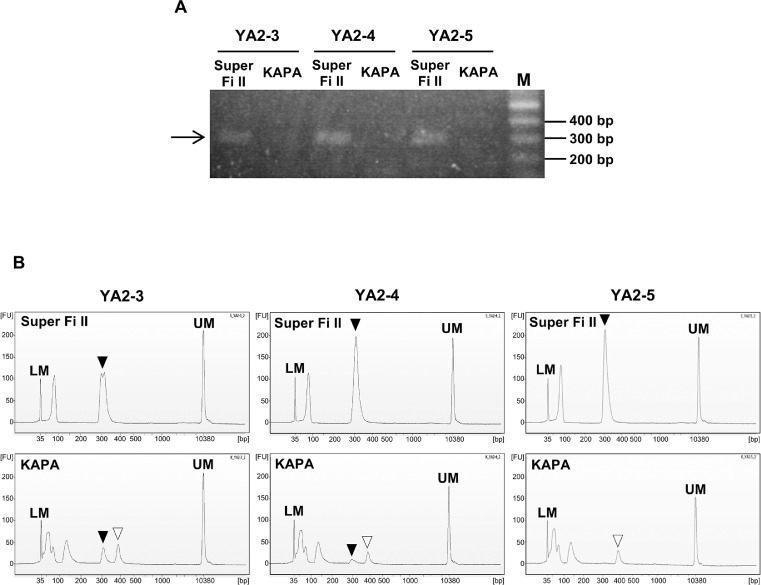
MiFish PCR amplicons from eDNA samples derived from pumped deep-sea water collected at the Yaizu site. Approximately 20 ng of eDNA was added in each 12 µL PCR mixture. A: Image of agarose gel electrophoresis. B: Electropherograms obtained by Bioanalyzer. X and Y-axes in electropherograms represent fragment size (bp) and fluorescence intensity (fluorescence units, FU), respectively. An arrow and solid triangles indicate bands and a peak representing MiFish-targeted amplicons, respectively. Open triangles indicate a peak representing nonspecific products. Primer and adapter dimers are seen as peaks following a lower marker peak in the electropherograms. M: Size marker. LM: Lower marker. UM: Upper marker. YA2-3, YA2-4, and YA2-5 represent sample numbers.

To verify the effectiveness of the SuperFi II PCR enzyme in eDNA metabarcoding of deep-sea fish, we conducted PCR experiments using MiFish primers. We used Yaizu samples in this experiment because in our preliminary experiments, the efficiency and specificity in MiFish PCR amplification, rather than the eDNA yield, were the principal limitation in Yaizu samples in a series of metabarcoding processes. Filtration volume of each sample was approximately 10 L (Table 4). The PCR efficiency and specificity of SuperFi II were compared with those of KAPA. The composition of reagents and thermal cycling conditions of the KAPA PCR were set according to the manufacturer's instructions and the eDNA manual [33] (Supplementary Tables 1 and 2). First, we conducted PCR amplifications to compare the efficiency of the two enzymes using three eDNA samples as template. PCR amplicons were verified by agarose gel electrophoresis and an Agilent 2100 Bioanalyzer using an Agilent High Sensitivity DNA Kit (Agilent Technologies, Santa Clara, CA, USA). Accordingly, PCR using SuperFi II resulted in the detection of correct-sized PCR amplicons (the target of MiFish primers with adapters: *ca*. 300 bp) (Fig. 1). However, correct-sized amplicons were not detected or scarcely detected in PCR reactions using KAPA (Fig. 1), and a nonspecific product was slightly detected in reactions using KAPA (Fig. 1B). This result showed that SuperFi II polymerase amplified the target region of MiFish from trace amounts of eDNA more effectively and sensitively than KAPA. Another possible explanation might be differences in the tolerance of enzymes to inhibitors. PCR inhibitors might have accumulated in eDNA solutions extracted from large amounts of pumped deep-sea water. As such, the high tolerance of SuperFi II for inhibitors might have potentially contributed to the successful amplification.

**Table 4:** NGS reads of MiFish PCR amplicons derived from pumped deep-sea water collected at Yaizu using two different enzymes applied in the first PCR. YA2-3, YA2-4, YA2-5, YA1-5, YA1-7, and YA1-16 represent sample numbers.

eDNA No.	Filtration volume (L)	eDNA added in 1st PCR (ng/12 µL PCR reaction)	PCR enzyme used in the first PCR	Ratio of fish reads to total assembled reads (%)
YA2-3	10.0	22.4	SuperFi II	94.2
YA2-4	10.0	22.0	SuperFi II	93.7
YA2-5	10.0	21.2	SuperFi II	91.6
YA1-16	7.6	48.0	KAPA	41.8
YA1-5	9.6	48.0	KAPA	43.1
YA1-7	12.4	67.2	KAPA	54.5

**Fig. 2:**
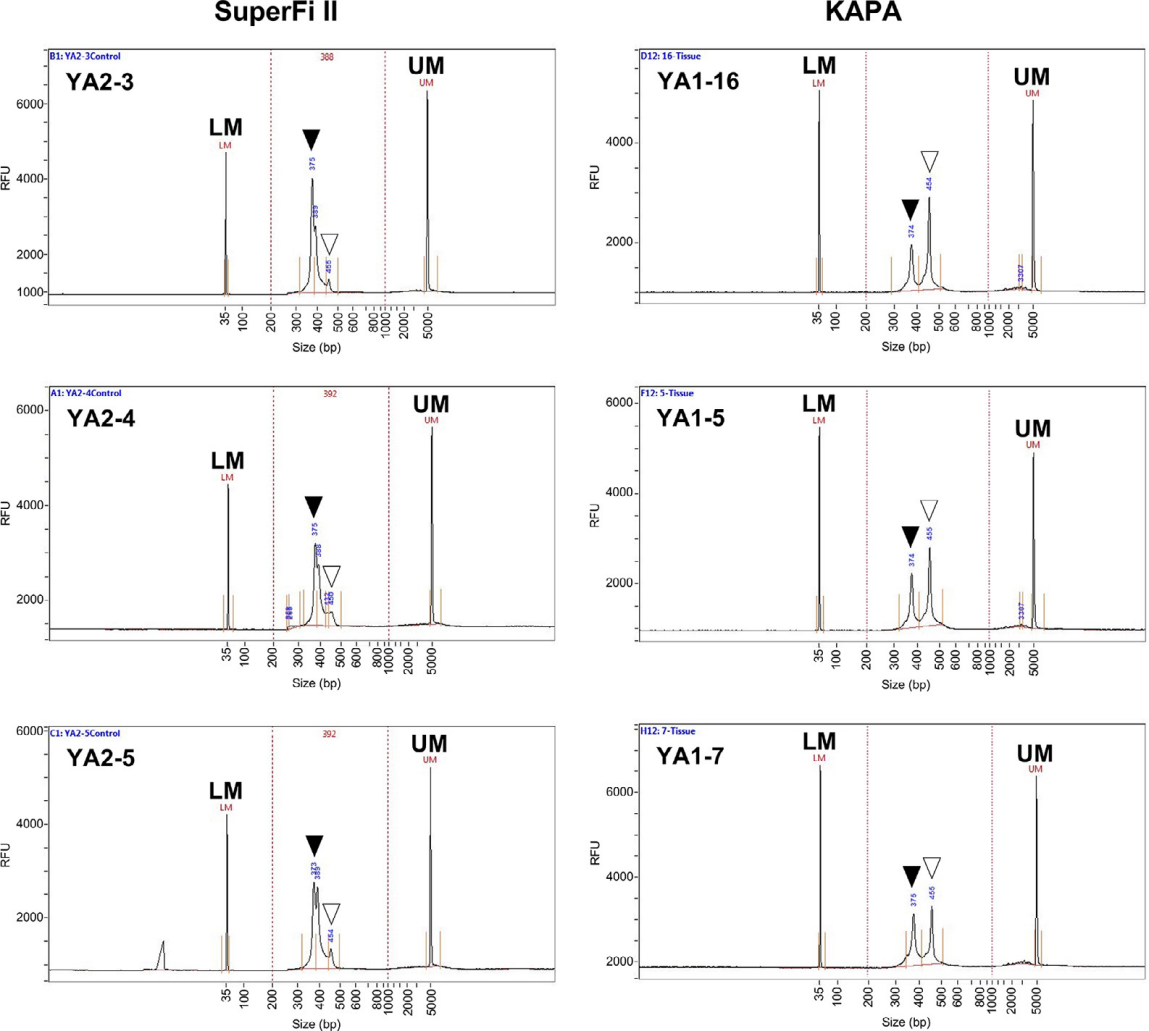
Electropherograms showing results of quality check of libraries constructed from MiFish PCR amplicons. X and Y-axes represent fragment size (bp) and fluorescence intensity (relative fluorescence units, RFU), respectively. Left panels (YA2-3, YA2-4, YA2-5) were amplified using SuperFi II and right panels (YA1-16, YA1-5, YA1-7) were amplified using KAPA in the first PCR. Solid triangles indicate a peak representing MiFish-targeted amplicons. Open triangles indicate a peak representing nonspecific amplicons. LM: Lower marker. UM: Upper marker. YA2-3, YA2-4, YA2-5, YA1-5, YA1-7, and YA1-16 represent sample numbers.

Subsequently, we performed the first PCR for the NGS library using MiFish primers. Three eDNA samples filtered at the Yaizu site were applied to each amplification using SuperFi II or KAPA (Table 4). After adjusting the amount of template eDNA, correct-sized PCR amplicons (*ca.* 300 bp) were acquired using both enzymes. However, an extra amplicon was detected only in the products obtained using KAPA, as seen in Fig. 1B (electrophoresis data not shown, collectively presented in the library results shown in Fig. 2). Using these PCR amplicons, we constructed three libraries per each enzyme used in the first PCR through the second PCR. Extraction of a target second PCR product from an excised band after gel electrophoresis, which is recommended in the eDNA manual, was not conducted in this study, because we compared the amplification specificity between Super Fi II and KAPA without removing nonspecific PCR products. The quality of libraries that were amplified by SuperFi II and KAPA in the first PCR were compared using an automated capillary electrophoresis Fragment Analyzer System with a dsDNA 915 Reagent Kit (Agilent Technologies, Santa Clara, CA, USA). A remarkable peak (*ca.* 370 bp), which was the target fragment from MiFish amplicons with adapters and index sequences, was acquired from the amplification using SuperFi II (Fig. 2). In contrast, using KAPA resulted not only in the acquisition of the target but also in the detection of an additional peak (Fig. 2), which was consistent with the first PCR results. This additional nonspecific PCR amplicon was presumed to be derived from the 16S rRNA gene of microorganisms and has been known to occur in PCR reactions using MiFish primers [28,33]. We assumed that the higher specificity of SuperFi II due to the superior hot-start technology enabled the suppression of this nonspecific amplification. The use of SuperFi II for the first PCR amplification of MiFish would thus have an advantage in eDNA metabarcoding of deep-sea fish using pumped deep-sea water that might potentially contain insufficient amounts of templates for PCR and certain amounts of PCR inhibitors and nontarget environmental microbial DNA.

Next generation sequencing by MiSeq was performed using libraries amplified by SuperFi II and KAPA. The results of sequence reads are presented below. BLASTN searches were performed against the MitoFish version 3.57 with reference sequences of 35,039 species (complete plus partial mitochondrial DNA data) [34,35]. The ratios of all assembled paired-end fish reads represented by identities greater than 97% are shown in Table 4. Fish ratios were less than 55% in KAPA amplified samples and approximately 93% in SuperFi II amplified samples (Table 4). Except for fish reads, other predominant reads were sequences of 16S rRNA genes that were nonspecific PCR amplicons derived from microorganisms, especially bacteria, in both cases. We therefore confirmed that MiFish PCR amplification using SuperFi II resulted in a low ratio of unnecessary NGS reads derived from microorganisms and effective acquisition of large amounts of information on fish eDNA through NGS. Most fish sequences obtained by NGS using both PCR enzymes were derived from deep-sea fish species inhabiting Suruga Bay at proper depths, such as *Pterothrissus gissu*, a species of genus *Paraliparis,Diaphus suborbitalis*, and *Hoplostethus japonicus*. The use of SuperFi II in the MiFish PCR was thus effective for eDNA metabarcoding of deep-sea fish.

### Other considerations

In a previous study, it was mentioned that enclosed filter cartridges, such as Sterivex had the advantage of reducing contamination risks from lab work by using cartridges without cutting the housing [20]. However, we employed the method of cutting the filter cartridge for our new extraction protocol for the following reason. Due to environmental conditions around the water intake port (see the graphical abstract), the pumped deep-sea water contains filtration clogging materials, such as sediment particles, resulting in a limited filtration volume (*ca*. 10 L at Yaizu, *ca*. 20 to 30 L at Akazawa) for each filter. We designed to construct each eDNA library using eDNA extracted from each filter cartridge filtered until clogging, so that we could collect as much metabarcoding data as possible. However, we could not acquire sufficient eDNA for MiFish amplification from each filter using the conventional method. Thus, we gave priority to the fragmentation of filters and sufficient immersion of fragments in lysis solution to increase eDNA yields. Meanwhile, we note that the opened cartridge method we presented here might increase contamination risks during the eDNA extraction process in the lab, as mentioned above. To prevent contamination with exogenous DNA after opening the cartridge, careful attention should be paid to the experimental environment, equipment, and operation, as described in the eDNA manual [21]. In this study, we prepared two extraction blanks and conducted eDNA extraction and MiFish PCR procedures using the blanks same as the other samples. We confirmed that no detectable DNA concentrations were measured in the extraction blanks and no PCR products were detected from blanks.

Our modified eDNA extraction protocol was thus specialized in collecting eDNA from deep-sea water, including only a few fish eDNA sources and certain amounts of PCR inhibitors. Additionally, in PCR and NGS experiments, we showed some comparative data as “representative results” of the employment of two distinct PCR enzymes in the case of the amplification of eDNA extracted from pumped deep-sea water. Comparison results presented here were not designed to demonstrate the superiority of this method over other reported methods in previous eDNA studies from various aquatic environments.

## Conclusion

To optimize eDNA metabarcoding of deep-sea fish using pumped deep-sea water, we proposed a protocol with minor modifications of conventional eDNA extraction and replacement of the PCR enzyme for MiFish PCR amplification. The modified eDNA extraction protocol enabled an increase in eDNA yields from pumped deep-sea water compared with the conventional method. SuperFi II could efficiently amplify the target region of MiFish from trace amounts of eDNA from pumped deep-sea water with suppressing nonspecific amplifications, eventually resulting in the acquisition of abundant sequences of deep-sea fish by NGS. The improved eDNA extraction and PCR amplification methods presented here could have a great impact on future advances in eDNA analyses of deep-sea fish.

## Declaration of Competing Interest

The authors declare that they have no known competing financial interests or personal relationships that could have appeared to influence the work reported in this paper.

## Appendix Supplementary materials

Image, application 1
