# Supplementary material for: Optimization of environmental DNA extraction and amplification methods for metabarcoding of deep-sea fish
Source: MethodsX. 2021 Jan 23;8:101238. doi: 10.1016/j.mex.2021.101238 (PMC8374181; doi:10.1016/j.mex.2021.101238)
Supplement: Supplementary file 1 [file mmc1.pdf]

Supplementary materials

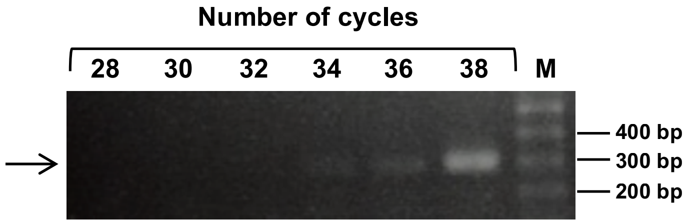

Supplementary Figure 1. The result of the preliminary experiment for the determination of the number of SuperFi II amplification cycles in MiFish PCR using eDNA from pumped deep-sea water. An arrow indicates MiFish-targeted bands. PCR conditions conformed to those described in Table 2 and in the manuscript. Approximately 30 ng of eDNA was added in each 12 μL PCR mixture. M: Size marker.

Supplementary Table 1

Composition of PCR reagents in KAPA DNA polymerase reactions (KAPA HiFi HotStart ReadyMix PCR Kit) for amplification of MiFish objectives using eDNA extracted from pumped deep-sea water. *1: This reaction includes 0.3 μM of MiFish-U and MiFish-E. *2: Volumes vary depending on the concentrations of reagents used.

Reagent components	Final concentration	Volume in 12 μL reaction
MiFish-Mix-F (Forward primer)	0.6 μM*1	—*2
MiFish-Mix-R (Reverse primer)	0.6 μM*1	—*2
Extracted eDNA	< 10 ng/μL	4 μL
2 × KAPA HiFi HotStart ReadyMix	1 ×	6 μL
Nuclease-free sterile water	—	to 12 μL

Supplementary Table 2

Thermal cycling conditions in KAPA DNA polymerase reactions (KAPA HiFi HotStart ReadyMix PCR Kit) for amplification of MiFish objectives using eDNA extracted from pumped deep-sea water. The annealing temperature was chosen in accordance with Miya *et al*. (2015) and the eDNA manual. The number of amplification cycles came from our preliminary experiment, same as the SuperFi II cycles described in the manuscript.

Step	Temperature	Duration	Cycles
Initial denaturation	95 °C	3 min	1
Denaturation	98 °C	20 s	
Annealing	65 °C	15 s	38
Extension	72 °C	15 s	
Final extension	72 °C	5 min	1
